# Activation of the interferon type I response rather than autophagy contributes to myogenesis inhibition in congenital DM1 myoblasts

**DOI:** 10.1038/s41419-018-1080-1

**Published:** 2018-10-19

**Authors:** Milena Rizzo, Pascale Beffy, Renata Del Carratore, Alessandra Falleni, Virginia Pretini, Romina D’Aurizio, Annalisa Botta, Monica Evangelista, Andrea Stoccoro, Fabio Coppedè, Denis Furling, Marcella Simili

**Affiliations:** 1 Institute of Clinical Physiology (IFC), CNR, Pisa, Italy; 2 Istituto Toscano Tumori (ITT), Firenze, Italy; 3 Department of Translational Research and New Technologies in Medicine and Surgery, University of Pisa, Pisa, Italy; 4 Department of Clinical and Experimental Medicine, Unit of Experimental Biology and Genetics, University of Pisa, Pisa, Italy; 5 Institute of Biophysics (IBF), CNR, Pisa, Italy; 6 Laboratory of Integrative Systems Medicine (LISM), Institute of Informatics and Telematics (IIT) CNR, Pisa, Italy; 7 Department of Biomedicine and Prevention, Medical Genetics Section, Tor Vergata University of Rome, Rome, Italy; 8 Department of Translational Research and New Technologies in Medicine and Surgery, Laboratory of Medical Genetics, University of Pisa, Pisa, Italy; 9 Sorbonne Université, INSERM, Association Institut de Myologie, Centre de Recherche en Myologie, F-75013 Paris, France

## Abstract

Congenital myotonic dystrophy type 1 (CDM1) is characterized by severe symptoms that affect patients from birth, with 40% mortality in the neonatal period and impaired skeletal muscle development. In this paper, we examined the relationship between autophagy and abnormal myogenic differentiation of CDM1 myoblasts. We investigated these pathological features at both ultrastructural and molecular levels, utilizing two CDM1 foetal myoblasts, CDM13 and CDM15, with 1800 and 3200 repeats, respectively. The congenital nature of these CDM1 myoblasts was confirmed by the high methylation level at the *DMPK* locus. Our results indicated that abnormal autophagy was independent of myogenic differentiation, as CDM13 myoblasts differentiated as well as control myoblasts but underwent autophagy like CDM15, displaying impaired differentiation. miRNA expression profiles revealed that CDM15 myoblasts failed to upregulate the complex network of myo-miRNAs under MYOD and MEF2A control, while this network was upregulated in CDM13 myoblasts. Interestingly, the abnormal differentiation of CDM15 myoblasts was associated with cellular stress accompanied by the induction of the interferon type 1 pathway (innate immune response). Indeed, inhibition of the interferon (IFN) type I pathway restores myogenic differentiation of CDM15 myoblasts, suggesting that the inappropriate activation of the innate immune response might contribute to impaired myogenic differentiation and severe muscle symptoms observed in some CDM1 patients. These findings open up the possibility of new therapeutic approaches to treat CDM1.

## Introduction

Myotonic dystrophy type 1 (DM1; MIM#160900) is a debilitating form of muscular dystrophy due to an abnormal increase of CTG repeats in the 3′ UTR region of the dystrophia myotonica protein kinase gene (*DMPK*). The disease is autosomal dominant, located on chromosome 19, and shows genetic anticipation, meaning that the disease symptoms tend to be more severe and to appear at an earlier age as the disorder is passed from one generation to the next. Indeed, both disease severity and age of onset correlate with the number of CTG repeats, ranging from about 100 to 1000 units in adult patients, and from 1000 to more than 3000 units in the congenital form (congenital myotonic dystrophy type 1 (CDM1)) that affects patients from birth and is characterized by severe hypotonia and respiratory insufficiency, with mortality reaching 40% in the neonatal period^1^. The mechanism by which the trinucleotide repeats expansion determines the complex phenotype of this disease has recently been partially clarified. One of the most accepted theories is that the CUG expansion induces abnormal nuclear accumulation of the mutant mRNA into foci, which in turn sequester the RNA-binding protein MBNL, thereby disrupting the normal alternative splicing and polyadenylation of many mRNAs involved in muscle cell metabolism (*trans*-dominant effect)^2^. However, splicing changes do not completely explain the DM1 phenotype, especially the congenital form, as they are shared by other muscular dystrophies, which do not have the same symptoms^3^. In a previous work, we found a profound impairment in the early steps of muscle differentiation of DM1 myoblasts with a high number of triplets, accompanied by major alterations of signal transduction pathways involved in differentiation^4^. Electron microscopy and molecular analysis revealed that these myoblasts were subject to increased autophagy after transfer into differentiating medium, suggesting that autophagy might be an escape mechanism for myoblasts unable to differentiate and partially responsible for muscle loss.

In this work, we investigated whether abnormal differentiation and autophagy could be related, and studied the molecular mechanisms that cause these pathological features using two congenital DM1 foetal myoblasts, CDM13 with 1800 and CDM15 with 3200 CTG repeats.

Our results indicated that aberrant autophagy is characteristic of both CDM1 myoblasts independently of their capacity to differentiate. miRNA profiles showed that CDM15 myoblasts with impaired myogenic differentiation failed to upregulate myo-miRNAs that are under the control of *MYOD* and *MEF2A*. We showed that an abnormal activation of the interferon (IFN) type 1 pathway in defective CDM15 myoblasts contributes to altered myogenesis.

## Results

### Increased autophagy characterizes both congenital DM1 myoblasts independently of their ability to differentiate

**Fig. 1:**
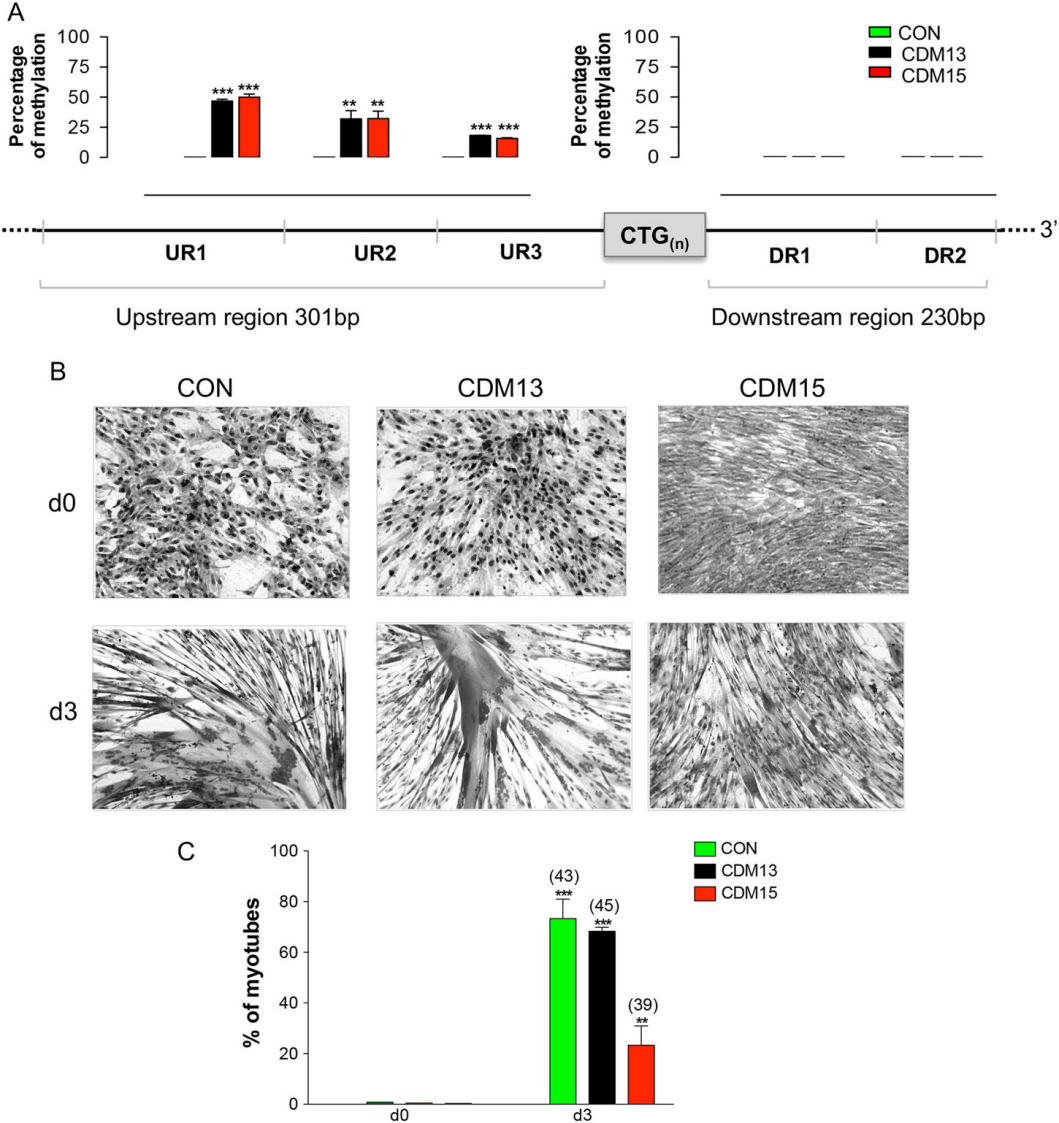
Characterization of CDM1 myoblasts. **a** High-resolution melting analysis of the *DMPK* locus. Percentage of methylation in the region upstream (UR1-UR3, left side) and downstream (DR1-DR2, right side) of the CTG repeats at the DMPK locus of CON, CDM13, CDM15 (upper panel), and schematic representation of the regions analyzed at the *DMPK* locus (lower panel). **b** Images of control (CON) and of the two CDM1 myoblasts, CDM13 (1800 repeats) and CDM15 (3200 repeats) after 3 days in differentiation medium (Giemsa staining). **c** Percentage of myotubes obtained by dividing the number of myotubes (cells with more than three nuclei) by the number of counted nuclei (at least 1000 nuclei for each time point). The average number of nuclei per myotube, evaluated by counting a total of 500 myotubes, is reported in brackets above each column

In this study, we used in vitro cell cultures of two DM1 foetal myoblasts (DM15 and DM13) with more than 1000 repeats, and control foetal myoblasts (CON). The repeats number of DM15 was 3200 CTG;^4^ DM13 was characterized by a combination of LR-PCR and TP-PCR analyses^5^ as having a 1800 CTG expansion without interruption at the 5′  or 3′ ends of the repeated array (Supplementary Fig. 1). As congenital forms of DM1 correlate with high levels of methylation of the CpG islands flanking the CTG repeats rather than simply the repeat number,^6,7^ we determined the methylation of these regions by the high-resolution melting (HRM) technique (Fig. 1a, Supplementary Fig. 2). High levels of methylation were found in the region upstream of the CTG repeats (UR1, UR2, and UR3 amplicons) of both DM13 and DM15 (see schematic representation in Fig. 1a), but no methylation was seen in CON. The amplicons downstream of the CTG repeats (DR1 and DR2) were hypomethylated in CON and DM1 myoblasts. The methylation and high number of CTG repeats indicate both DM1 myoblasts to be congenital and they are henceforth designated as CDM13 (1800 repeats) and CDM15 (3200 repeats).

**Fig. 2:**
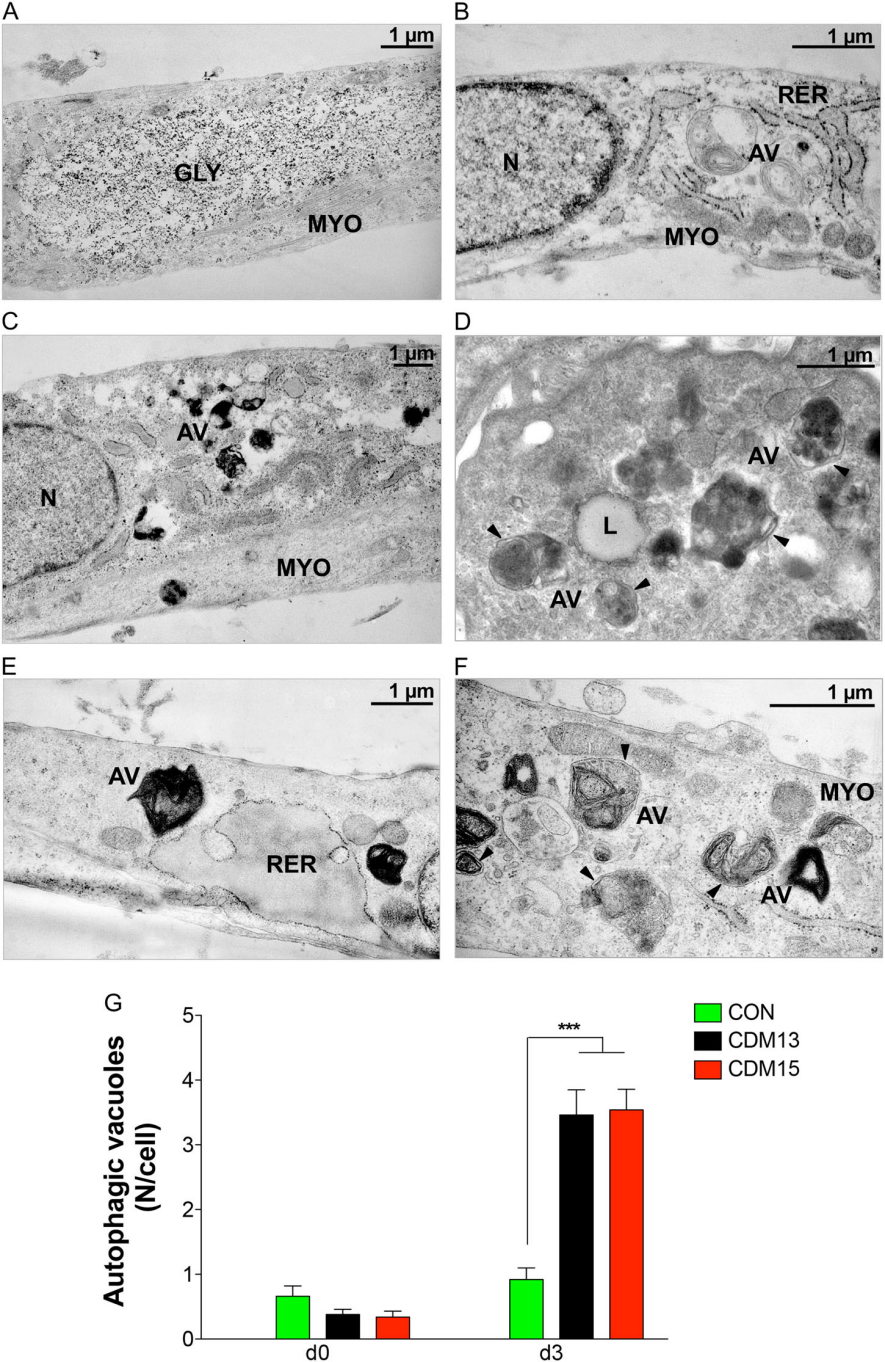
Analysis of autophagic vacuoles by transmission electron microscopy. Representative micrographs of autophagic vacuoles (AV) in CON (**a**, **b**), CDM13 (**c**, **d**), and CDM15 myoblasts (**e**, **f**) after 3 days in differentiation medium. **g** Average number mean (±SE) of AV/cell determined by counting the AV in at least 50 myoblasts. The significance was calculated with the multifactor analysis of variance, MANOVA (***p < 0.001). GLY glycogen particles, MYO myofibers, AV autophagic vacuoles, N nucleus, RER rough endoplasmic reticulum. Arrow heads indicate the vacuole double membranes

**Fig. 3:**
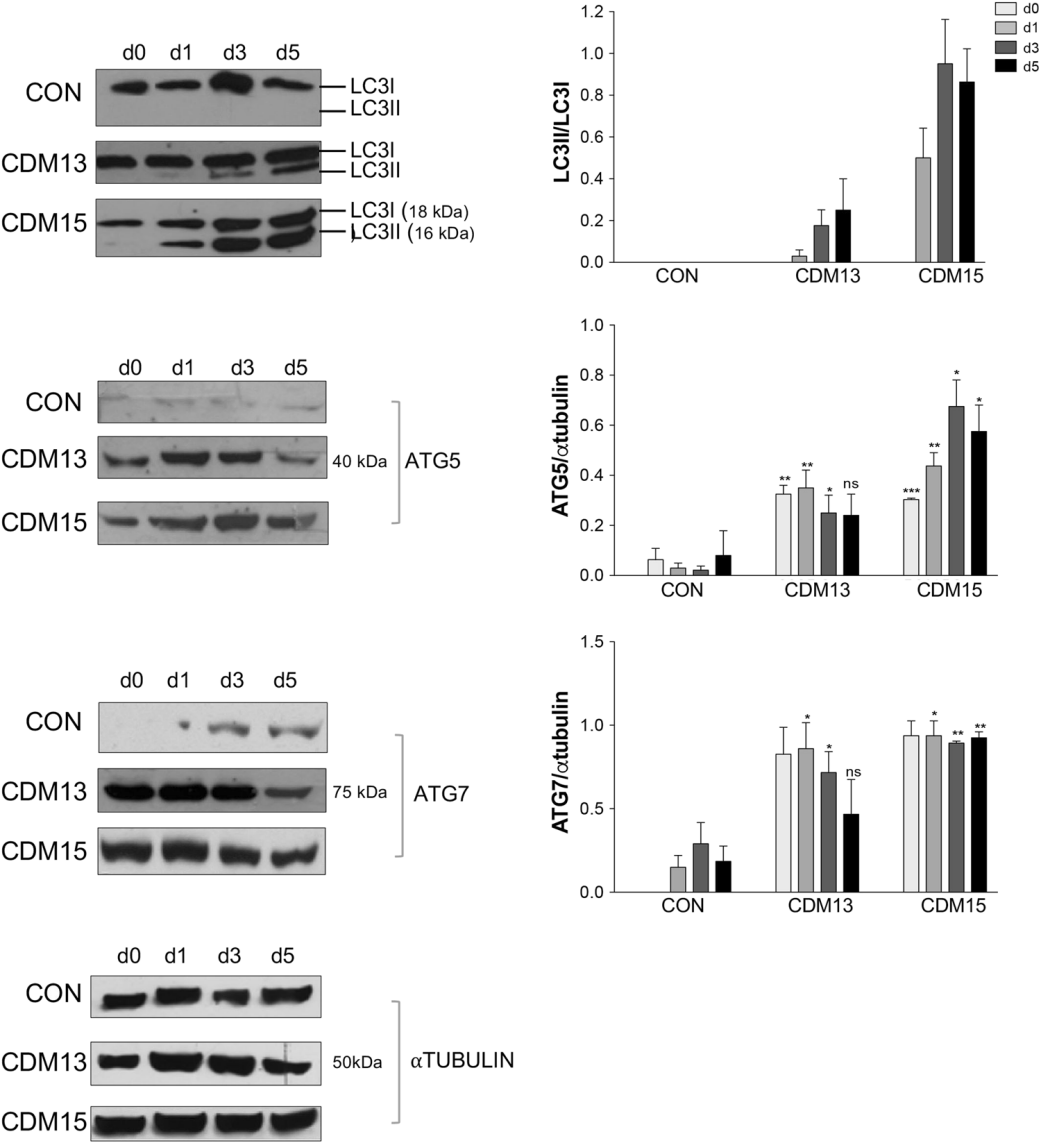
Analysis of proteins involved in autophagy. Representative LC3, ATG5, and ATG7 protein quantifications by immunoblot analysis of CON, CDM13, and CDM15 myoblasts before and after exposure to differentiation medium (days 0–5). LC3 activation was determined by calculating the ratio between the cleaved active LC3-II form and the non-active LC3-I form. The levels of ATG5 and ATG7 were normalized to that of αtubulin. All data were reported in the corresponding histogram. Results are expressed as mean (SD) and data analyzed by Student’s t-test (*P < 0.05, **P < 0.01, ***P < 0.001). The significance was calculated in CDM13 or CDM15 versus CON for each day

Myoblasts were induced to differentiate by shifting sub-confluent cultures into differentiating medium: CDM13 differentiated as well as CON (about 70% and 80% of myotubes, respectively, at day 3), while CDM15 myoblasts differentiated poorly (about 30% of myotubes) (Fig. 1b, c). We previously hypothesized that autophagy activation could be the consequence of abnormal differentiation;^4^ for this reason, CDM13, CDM15, and CON myoblasts were analyzed by electron microscopy. We found a low level of autophagy in all analyzed myoblasts both at day 0 and day 3 (Fig. 2g). However, under differentiating conditions (day 3), autophagy increased significantly in both CDM1 myoblasts compared to CON (Fig. 2a–f), measured as the number of autophagic vacuoles per cell in both well-differentiated CDM13 and poorly differentiated CDM15 myoblasts (Fig. 2g). This is clearly visible in Fig. 2c, where CDM13 myoblasts showed an abundance of myofibrils, concomitant with numerous autophagic vacuoles. These results do not support the hypothesis of a causative association between autophagy and abnormal differentiation^4^. Some vacuoles have the typical double membrane characteristic of autophagosomes (Fig. 2d, f, black arrow heads), while other more mature autophagosomes, which contain black material, do not show double membranes^8^. The small increase of AV diameter observed at day 3 in comparison to day 0 in all types of myoblasts (Table 1) could be a reflection of a physiological increase of autophagy, necessary for differentiation as reported by Fortini et al.^9^. However, LC3 activation and a significant ATG5/ATG7 upregulation were found in CDM13/CDM15 shifted to differentiation medium, but not in CON (Fig. 3).

**Table 1:** Diameter (mean±SD) of autophagic vacuoles (AV)

	AV diameter (µm)
CON	
Day 0	0.68 ± 0.23
Day 3	1.13 ± 0.32
CDM13	
Day 0	0.90 ± 0.25
Day 3	1.33 ± 0.55
CDM15	
Day 0	0.66 ± 0.33
Day 3	1.16 ± 0.44

**Fig. 4:**
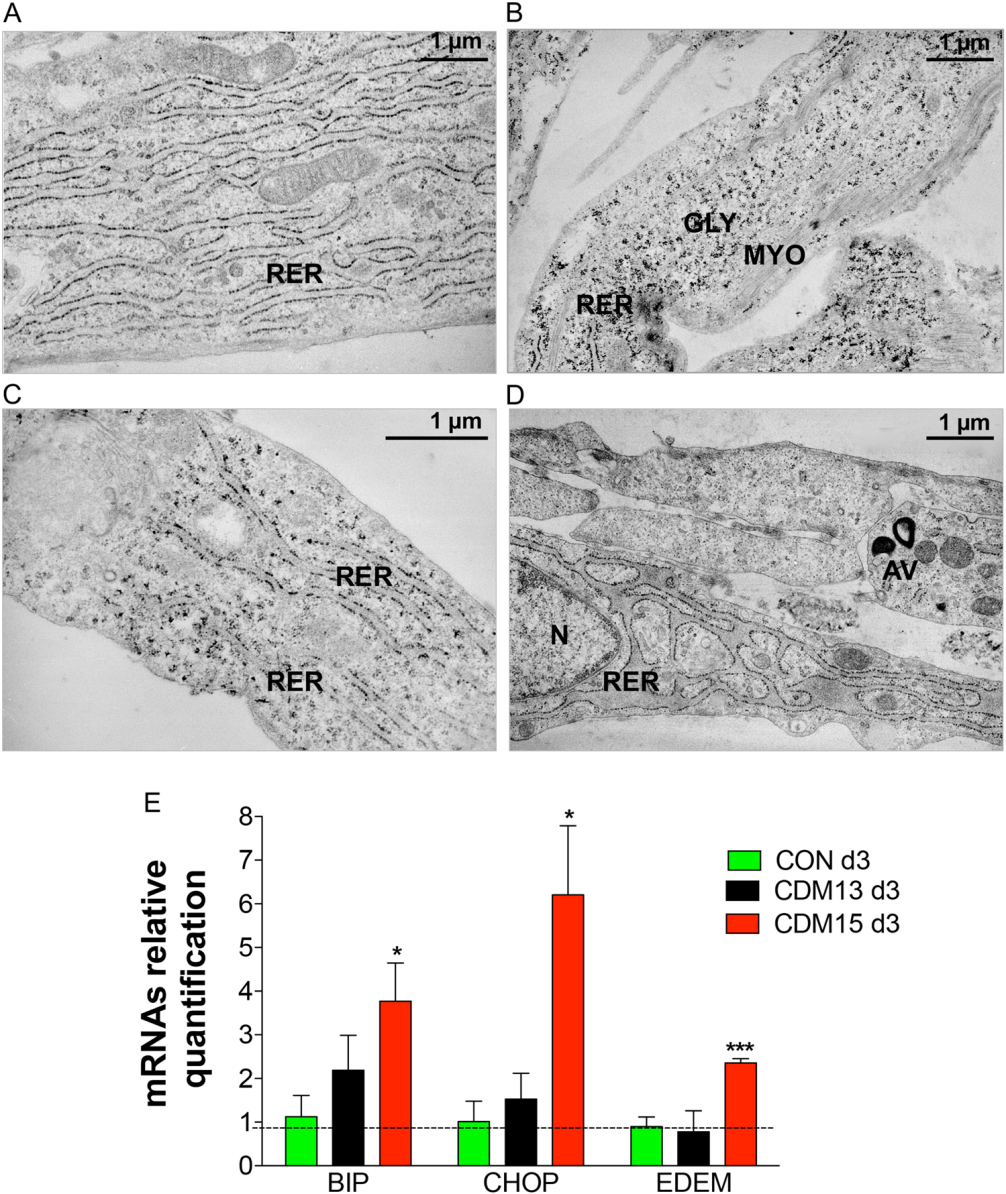
Analysis of the rough endoplasmic reticulum (RER). Representative micrographs of normal RER observed with transmission electron microscopy at day 3 in CON (**a**, **b**) and CDM13 (**c**), and of very enlarged RER present in CDM15 myoblasts (**d**). GLY glycogen particles, MYO myofibers, AV autophagic vacuoles, N nucleus, RER rough endoplasmic reticulum. **e** Relative BIP, CHOP, EDEM mRNA expression levels, quantified by qRT-PCR, in CON, CDM13, and CDM15 on day 3. Data were normalized using the geometric mean of three reference genes (*GUSB*, *TBP*, and *RPS18*). Results are expressed as mean (SD) of at least three independent experiments and data analyzed by Student’s t-test (*P < 0.05, **P < 0.01, ***P < 0.001). The significance was calculated in CON, CDM13, or CDM15 day 3 versus the corresponding day 0

An abnormal rough endoplasmic reticulum (RER) with enlarged cisternae, indicative of cellular stress,^10^ was found in CDM15 myoblasts at day 3 (Fig. 4d), unlike either CDM13 or CON, showing normally shaped RER (Fig. 4a–c). This result was confirmed by analysis of the ER stress molecular markers BIP, CHOP, and EDEM (Fig. 4e), whose mRNA levels only increased significantly at day 3 in CDM15 cells.

### Altered expression of MYOD, MEF2A, and muscle differentiation-related miRNAs in CDM15 myoblasts

To identify molecular pathways involved in defective myogenic differentiation of CDM15 myoblasts, we profiled global expression of miRNAs (miRNome, data available at GEO NCBI, GEO accession number GSE97019) of CON, CDM13, and CDM15 myoblasts before (day 0) and after 3 days in differentiating medium (day 3). The overall relationship among the miRNomes was investigated by PCA analysis (Supplementary Fig. 3), which indicated that CDM13 and CON miRNomes were similar to one another but differed from CDM15 miRNomes, especially at day 3.

**Fig. 5:**
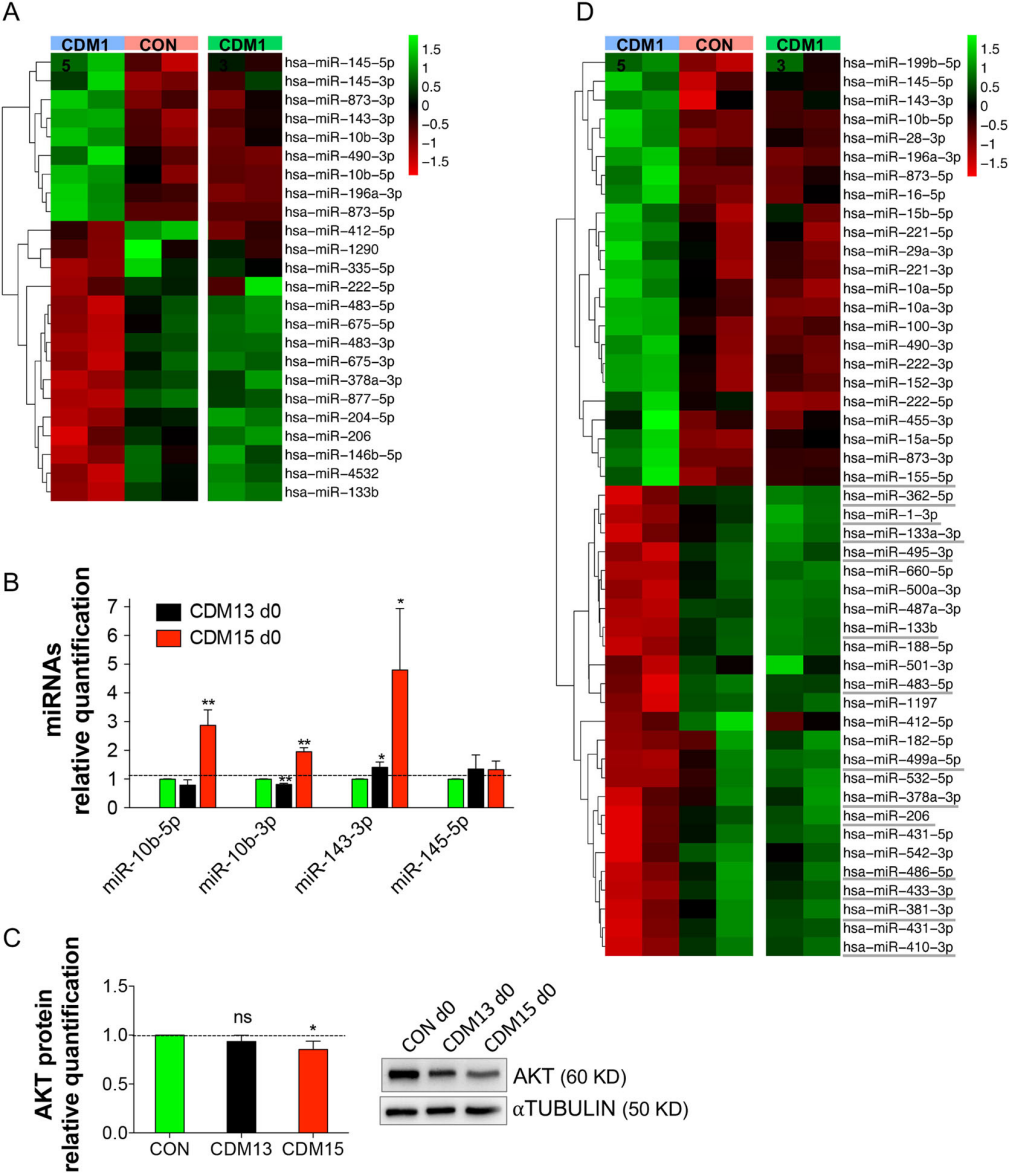
miRNA profile and validation. Heat map of miRNAs differentially expressed in CDM15 versus CON at day 0 (CDM15vsCON(d0)-miRNAs) (**a**) and at day 3 (CDM15vsCON(d3)-miRNAs) (**d**): the expression level of these miRNAs was also checked in CDM13 (third column). Relative miR-10b-5p, -10b-5p, -143, -145-5p (**b**) and AKT protein (**c**) expression levels in CON, CDM13, and CDM13 at day 0 . Data were normalized using *U6* or αtubulin for miRNAs and AKT protein, respectively. Results are expressed as mean ± SD of at least three independent experiments and data analyzed by Student’s t-test (*P < 0.05, **P < 0.01, ***P < 0.001) . The significance was calculated in CDM13 or CDM15 day 0 versus CON day 0

We analyzed miRNAs differentially expressed at both day 0 (Fig. 5a, Supplementary Table 1) and day 3 (Fig. 5d, Supplementary Table 2) in CDM15 versus CON and found 24 miRNAs (9 upregulated and 15 downregulated miRNAs, |log2FC| > 1, padj ≤ 0.05) at day 0 (named CDM15vsCON(d0)-miRNAs), and 48 miRNAs (23 upregulated and 25 downregulated miRNAs, |log2FC| > 1, padj ≤ 0.05) at day 3 (named CDM15vsCON(d3)-miRNAs). We also checked the expression levels of CDM15vsCON(d0)- and CDM15vsCON(d3)-miRNAs in CDM13 at day 0 (Fig. 5a, third column) and day 3 (Fig. 5d, third column) miRNomes and found that the expression level of CDM15vsCON-miRNAs in CDM13 was comparable to that found in CON, suggesting that the miRNAs altered in CDM15 were normally expressed in CDM13.

To clarify the possible functional role of CDM15vsCON-miRNAs we examined the pathways in which their target genes are involved.

For the CDM15vsCON(d0)-miRNAs, we focused only on the validated targets from well-annotated databases (e.g. MirTarBase, miRecords) and found that 124 KEGG pathways were significantly enriched (*p* ≤ 0.05, Supplementary Table 3). Interestingly, some of the CDM15vsCON(d0)-miRNA targets belonged to pathways necessary for differentiation. Thus, the upregulation of miR-10b-5p and miR-143-3p, which target *PI3K* and *IGF1R/AKT*, respectively, as well as that of miR-10b-3p and miR-145-5p, which target *IGF1R* (Fig. 5b), could contribute to the inhibition of the mTOR pathway, whose activation is required for differentiation^11^ (Supplementary Table 4). Indeed, the AKT level of CDM15 at day 0 is slightly but significantly lower than in control myoblasts, supporting the hypothesis that miR-143-3p upregulation might be involved in AKT decrease (Fig. 5c). In accordance with this result, p-S6K1 phosphorylation level is lower in CDM15 both at day 0 and day 3 in comparison to CON and CDM13 (Supplementary Fig. 4) as previously shown^4^. It is worth noting the miR-206, required for differentiation^12,13^, was downregulated in CDM15 myoblasts compared to CDM13/CON myoblasts, supporting the hypothesis of an early commitment of CDM13 toward differentiation that is lacking in CDM15 myoblasts.

**Fig. 6:**
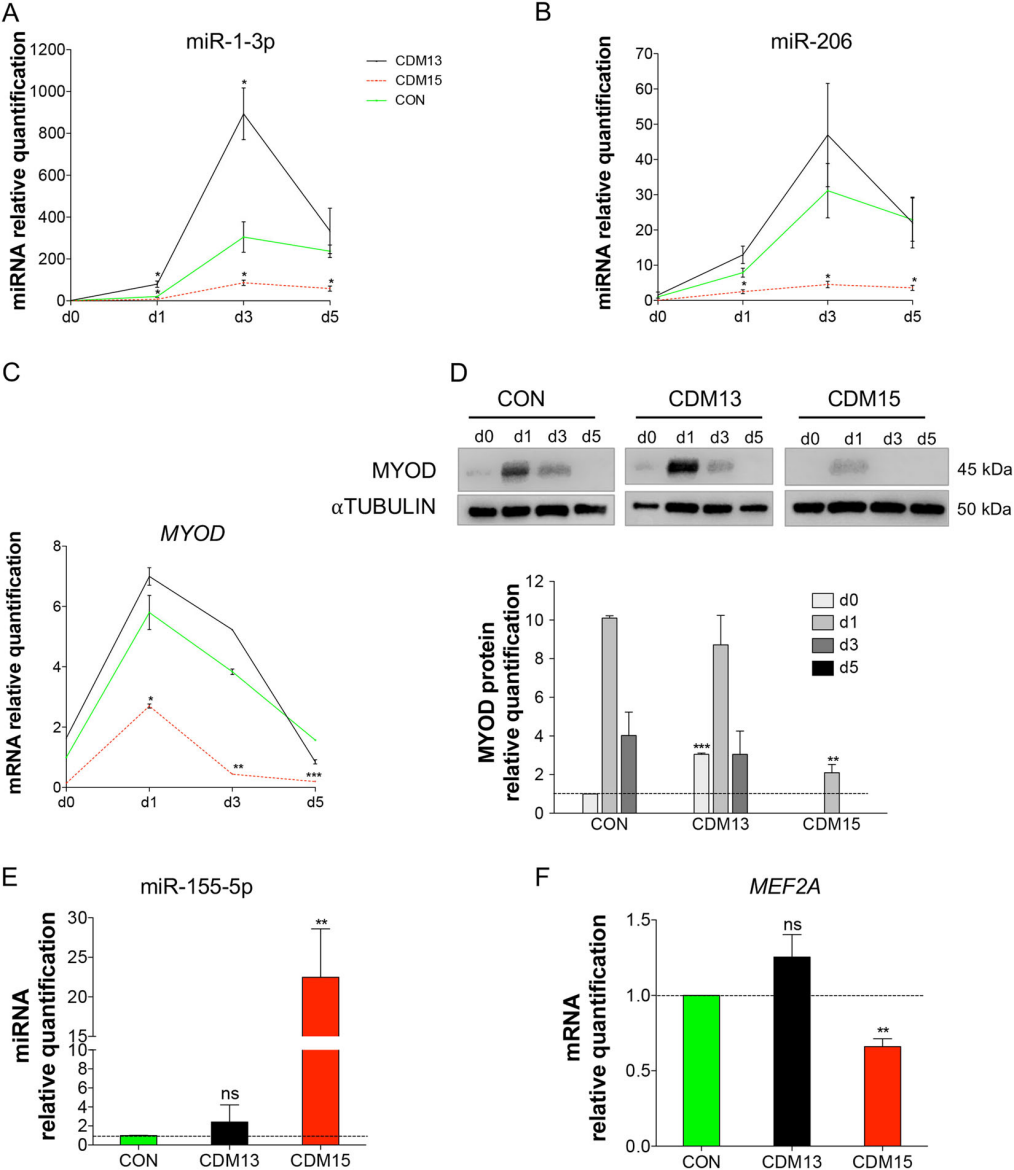
Expression analysis of miRNAs and genes related to differentiation. Relative expression level quantification determined by qRT-PCR of miR-1-3p (**a**), miR-206 (**b**), MYOD mRNA (**c**), and protein (**d**) during the exposure to differentiation medium (days 0––5) and of miR-155-5p (**e**) and MEF2A (**f**) at day 3. The relative expression was evaluated with respect to CON at day 0 for (**b**–**d**) and with respect to CON at day 3 for (**e**) and (**f**). Data were normalized using *U6*, αtubulin or the geometric mean of three reference genes (*GUSB*, *TBP* and *RPS18*) for miRNAs, proteins and *MYOD*/*MEF2A* mRNAs respectively. Results are expressed as mean ± SD of at least three independent experiments and data analyzed by Student’s t-test (*P < 0.05, **P < 0.01, ***P < 0.001). The significance was calculated in CDM13 or CDM15 versus CON for each day

As expected, many of the CDM15vsCON(d3)-miRNAs play a major role in muscle differentiation (the so-called myo-miRNAs, Fig. 5d, underlined miRNAs)^12^. We validated two important myo-miRNAs, miR-1-3p, and miR-206, by qRT-PCR (Fig. 6a, b): as expected, both miRNAs were poorly induced in CDM15, while they progressively increased in CON/CDM13, reaching a peak at day 3. Since these miRNAs are regulated by MYOD^12,13^, we verified the relative MYOD expression. As expected (Fig. 1), MYOD was poorly induced in CDM15 (Fig. 6c, d) while it increased in both CDM13 and CON myoblasts with a time course matching that of miR-1-3p and miR-206. To investigate whether other deregulated miRNAs could play a role in myogenesis, we identified the validated targets of all CDM15vsCON(d3)-miRNAs and searched for those belonging to the myogenesis pathway (id: R-HSA-525793) present in the REACTOME db (http://www.reactome.org/) (Supplementary Table 5). This approach highlighted the downregulation of miR-433, miR-495-3p, and miR-410-3p belonging to the Gtl2-Dio3 miRNA mega cluster, fundamental for skeletal muscle regeneration^14^ and controlled by *MEF2A*, and the upregulation of miR-155-5p, known to target *MEF2A*^15^. Indeed, we found that *MEF2A* was significantly downregulated (Fig. 6f), while miR-155-5p was upregulated (Fig. 6e), at day 3 in CDM15 versus CON/CDM13. Overall these results indicated that the expression of a number of miRNAs involved in myogenesis was impaired in CDM15 myoblasts.

### Activation of IFN type 1 response in CDM15 myoblasts

Analysis by electron microscopy revealed that CDM15 myoblasts showed not only autophagic vacuoles but also a remarkably stressed RER (Fig. 4d) compared to CDM13/CON. p53 increase^16^ in CDM15 (Supplementary Fig. 5) also suggested the presence of a marked cellular stress in these myoblasts not present in CDM13. Different toxic stimuli could be the basis of CDM15 cellular stress: it has been shown that toxic RNAs containing CUG repeats accumulate in the nuclei of DM1 myoblasts (foci)^2,17^, and that this RNA can give rise to dsRNA structures^18^. It is known that dsRNA elicits IFN type 1 (IFN1) production, which activates the innate immune response in an autocrine fashion^19^. We hypothesized that dsRNA structures, produced by transcription of the mutant *DMPK* allele, might induce an IFN response, as previously proposed^20^, and inhibit muscle differentiation^21^.

**Fig. 7:**
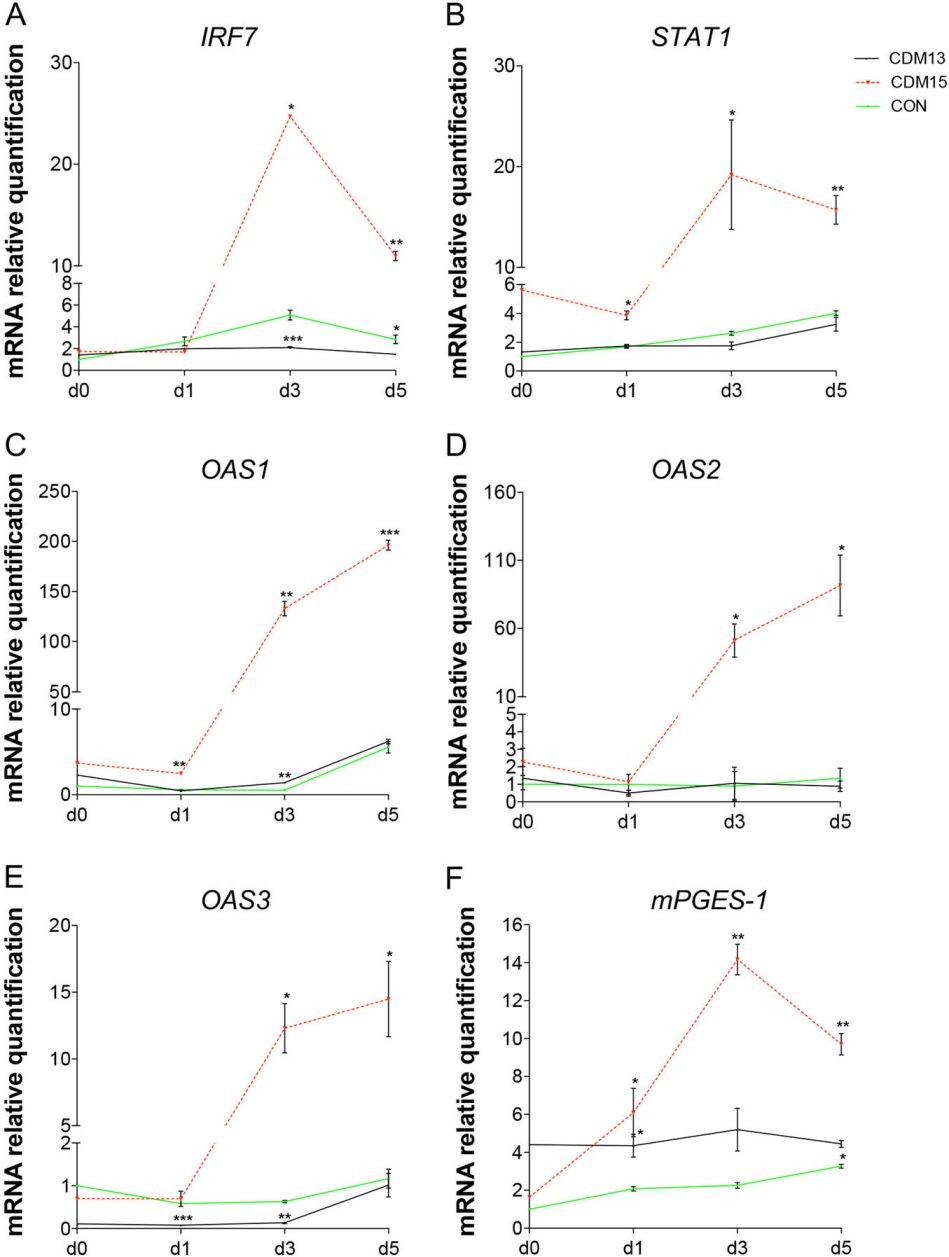
Analysis of interferon type 1 pathway. Relative expression level quantification of *IRF7* (**a**), *STAT1* (**b**), *OAS1* (**c**), *OAS2* (**d**), *OAS3* (**e**), and *mPGES-1* (**f**) after the exposure to differentiation medium (days 0–5). The relative expression was evaluated with respect to CON at day 0. Data were normalized and analyzed as described in figure 6, and were expressed as mean ± SD of at least three independent experiments

For this reason we analyzed *IRF7*, one of the first induced IFN1-positive regulators^22^, as well as some of the IFN1-upregulated genes such as *STAT1*^23^, and the three 2′–5′ oligoadenylate synthetase genes (*OAS 1*, *OAS 2*, and *OAS 3*)^24^, and found that their levels markedly increased in CDM15 versus either CON or CDM13 myoblasts (Fig. 7a-e). Finally, as IFN1 is known to induce the release of inflammatory cytokines^25^, we measured *mPGES-1* and found a marked increase in CDM15 myoblasts shifted to differentiation medium (Fig. 7f), as previously published^26^. Altogether, these results suggest that CDM15, upon exposure to differentiation medium, undergo a marked upregulation of the IFN1 pathway, which could be directly or indirectly causatively correlated with the inhibition of differentiation observed in these myoblasts.

It has recently been reported that an increase of the sense *DMPK* transcription coupled with the reduction of *DMPK* antisense transcription was responsible for the enhanced production of toxic dsRNA formation in patients with severe CDM^27^. We checked the levels of the two transcripts in all three myoblasts and found that the level of *DMPK* sense transcript appeared to be higher than that of the antisense transcript in CDM13/CDM15 myoblasts (day 3), with the ratio sense/antisense being very similar in both CDMs and higher than in control myoblasts (Supplementary Fig. 6). These results, while in keeping with the hyper-methylation of the upstream CTG region in CDM1 patients, do not explain the different degree of differentiation observed in CMD13/CDM15 myoblasts.

**Fig. 8:**
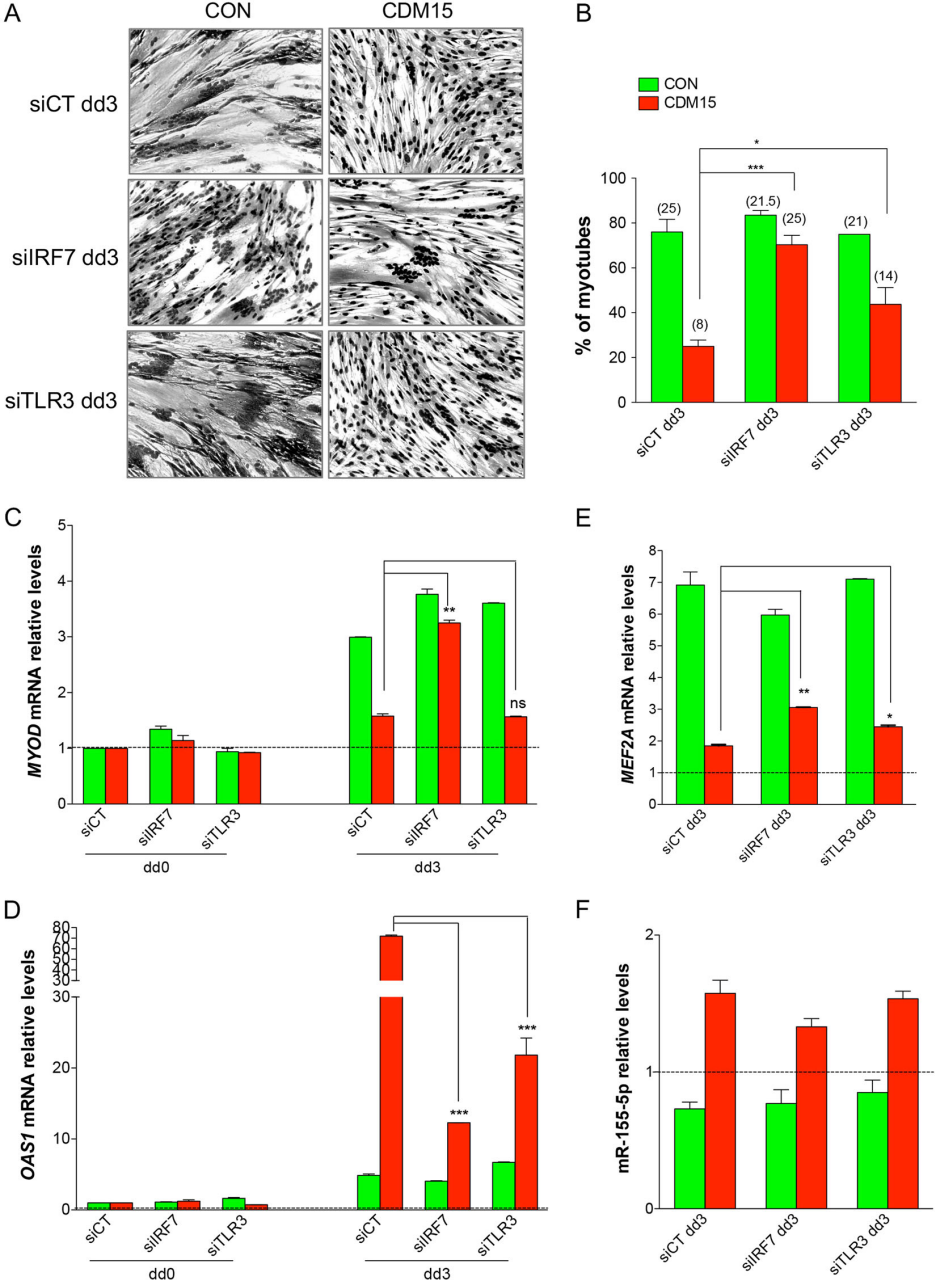
**Myogenic differentiation after IRF7 and TLR3 silencing.** (**a**) Images of CON, CDM13, and CDM15 myoblasts exposed to three days of differentiation medium after IRF7 (siIRF7), TLR3 (siTLR3), and control (siCT) silencing (dd3) (Giemsa staining). (**b**) Percentage of myotubes and average number of nuclei per myotube in CON and CDM myoblasts at dd3 evaluated as described in Fig. 1. Relative expression level of MYOD (**c**), OAS1 (**d**), MEF2A (**e**), and miR-155-5p (**f**) in CON, CDM13, and CDM15 myoblasts dd3 versus the respective dd0 (dd0 is the time point after the end of transfection and before the exposure to differentiation medium). Normalization and statistical analysis were done as described in Fig. 6. The significance was calculated in siIRF7 dd3 and siTLR3 dd3 samples versus siCT dd3

As the upregulation of the IFN1 pathway in CDM15 myoblasts correlated with impaired myogenesis, we silenced two genes known to control this pathway at two different levels: (i) Toll-like receptor 3 (TLR3), which is part of a family of receptors^28^ that recognize dsRNA and as such controls one of the early steps of the pathway; (ii) IRF7, considered to be one of the master regulators of IFN1, acting downstream of TLR3^29^ and markedly upregulated in CDM15 at day 3 (Fig. 7a). We transfected CDM15/CON myoblasts with siRNAs against IRF7 or TLR3 and then shifted them in differentiating medium for 3 days: at this time point the IRF7 and TLR3 mRNA levels were strongly reduced (Supplementary Fig. 7). The silencing of IRF7 and, to a lesser extent, TLR3 partially rescued the defective CDM15 differentiation as shown by the significant increase of myotubes formation in terms of number of myotubes and nuclei per myotube (Fig. 8b) at day 3. Restored myogenesis of CDM15 myoblasts was accompanied by an increase of *MYOD* (Fig. 8c) and *MEF2A* (Fig. 8e) and a strong decrease of *OAS1* (Fig. 8d). We also tested the miR-155-5p levels, as this miRNA could be under the control of IFN1. Although miR-155-5p tended to decrease after TLR3 or IRF7 silencing in CDM15 myoblasts, the variations were not significant (Fig. 8f); this suggests that in this context miR-155-5p is neither controlled by IFN1 nor controls *MEF2A*.

## Discussion

We had previously shown that CDM15 foetal myoblasts with high number of repeats (> 3000) did not differentiate properly and underwent autophagy, but the molecular mechanisms underlying this behavior were not fully investigated, nor was it clear whether autophagy was causatively related to aberrant differentiation^4^. To better investigate these aspects we included in our study another congenital foetal DM1 myoblast, CDM13, containing 1800 repeats and found that their behavior, concerning differentiation, were totally different: CDM15 differentiated poorly (as previously reported^4^), whereas CDM13 differentiated like control myoblasts.

Because miRNA expression plays a major role in muscle development and dysfunction^12^, a global miRNA expression profile (miRNome) was performed for the first time on foetal CDM1 and control myoblasts.

The miRNA profiles showed a severe deregulation of the miRNAs, that control myogenesis (myo-miRNAs), reflecting the defective differentiation observed in CDM15 myoblasts. Moreover, miRNA deregulation might be an early event present at the time of commitment toward differentiation since some of the miRNAs deregulated at day 0 in CDM15 can predict the inhibition of the differentiation program. For instance, miR-10b-5p, miR-145-5p, and miR143-3p, which target important mediators of the mTOR pathway essential for myogenesis^11^, were upregulated, while miR-206, one of the most important myo-miRNAs^12,13^, was downregulated. As expected, the most significant differences between CDM15 and CDM13/CON were found at day 3, when the differentiation program is fully activated and many of the so-called myo-miRNAs were induced in the two types of differentiating myoblasts (CON/CDM13) but not in CDM15 (Fig. 5d). In line with these results, CDM15 myoblasts shifted to differentiation medium were unable to upregulate MYOD, one of the master myogenic transcription factors that controls miR-1-3p, miR-133a-3p, miR-133b, and miR-206 expression. Interestingly, Amack et al.^30^ reported that defective myogenic differentiation of C2C12 murine myoblasts expressing expanded CTG repeats was associated with an absence of MYOD at the protein level although *MYOD* mRNA expression was normal. On the contrary, we found that in CDM15 MYOD was poorly induced both at the protein and mRNA levels, possibly due to increased mRNA decay, although inhibition of transcription cannot be ruled out.

Some of the miRNAs (Fig. 5d) belonging to the Gtl2-Dio3 miRNA mega cluster, fundamental for skeletal muscle regeneration^14^ and controlled by MEF2A, were downregulated, suggesting that, along with MYOD, the MEF2A transcription network of CDM15 myoblasts might also be altered. Finally, we wish to point out that 10 out of 23 upregulated miRNAs in CDM15vsCON(d3) (miR-199a, -145, -143, -28, -221-3-p, -221-5-p, -222-3p, -222-5p, -100, -155) were also found upregulated in many primary muscular disorders including Duchenne muscular dystrophy^31^.

Our data indicate that a marked increased autophagy and aberrant differentiation are not necessarily connected. Indeed, CDM13, which differentiated as well as control myoblasts, underwent increased autophagy to the same extent as CDM15 myoblasts. We wish to point out that in our system two levels of autophagy induction were evident: a physiological and an aberrant one. In all tested myoblasts, we found autophagic vacuoles at day 0 undergoing an enlargement by day 3, indicating that a certain increase of autophagy takes place as a “physiological” mechanism necessary for differentiation^9^. As far as LC3 activation is concerned we did not observe any activation in CON myoblasts at day 3, as previously reported in another human control foetal myoblast^4^. LC3 activation was only visible in CDM1 myoblasts concomitant with an abnormal increase of autophagy, which appears to be independent from differentiation as it also occurs in aberrantly differentiating CDM15. This pathological increase of autophagy could contribute to the marked reduction of muscle mass observed in CDM1 patients, a feature that has been also reported in DM1 patients^32^.

Finally, our study indicated that the abnormal differentiation observed in CDM15 myoblasts was associated with p53 upregulation, the presence of a stressed RER, and the activation of the IFN1 pathway. The findings that the IFN1-positive regulator IRF7, the downstream effector STAT1 and the IFN1-regulated genes *OAS 1*, *OAS 2*, and *OAS 3*, increased many fold in CDM15 compared to CON/CDM13 indicated a marked upregulation of IFN1 pathway (innate immune response). The product of the induced OAS enzymes is the small oligonucleotide 2′–5′ oligo A, which in turn activates the latent endoribonuclease RNase L, leading to degradation of RNA including cellular mRNAs^33^. Therefore, the activation of the interferon pathway and the consequent upregulation of the *OAS* gene family in CDM15 myoblast, could be responsible for the poor induction of *MYOD* mRNA. Interestingly, the activation of the 2′–5′ oligoadenylate/RNase pathway has been shown to delay C2C12 myoblasts differentiation via degradation of *MYOD* mRNA^34^.

Activation of interferon-regulated genes has been observed in lens epithelium from DM1 patients^35^. These authors hypothesized that toxic dsRNAs containing expanded CUG repeats might trigger the IFN1 response, analogously to RNA viruses. Although mutant mRNA is usually localized in foci within the nucleus of DM1 cells^2,17^, these foci undergo dynamic changes during the cell cycle (mitosis), resulting in cytoplasmic localization^20^ where dsRNA could be detected by dsRNA-specific receptors (such as the Toll-like receptor 3, TLR3)^36^ and induce the IFN1 pathway. As the activation of IFN1 response in CDM15 appeared to be causatively correlated to myogenesis inhibition, we tried to inhibit this response by silencing two genes acting at different levels of the IFN1 pathway, namely TLR3 and the downstream master regulator of IFN1 IRF7^29^, strongly upregulated in CDM15. Interestingly, the transient silencing of the two genes before shifting CDM15 into differentiating medium significantly ameliorated myogenesis, measured as the numbers of myotubes, without detectable effects in CON myoblasts. It is noteworthy that IRF7 silencing induced a number of myotubes comparable to control and a significant increase of *MYOD* and *MEF2A* mRNAs, the two transcription factors essential for myogenesis; concomitantly, there was a drastic reduction of OAS1, as expected if IFN1 was inhibited. These data confirm the powerful contribution of the IFN1 pathway activation to the impairment of myogenesis in CDM15 myoblasts. However, while the silencing of both IRF7 and TLR3 appear to be efficient, the rescue of differentiation was more pronounced after IRF7 inhibition. A possible explanation is that TLR3 is not the only member of the Toll-like receptors family responding to dsRNA^37^ and hence its inhibition may not result in the total reduction of dsRNA sensors. Finally, the inhibition of the IFN1 pathway did not significantly change miR-155-5p level, suggesting that in this context this miRNA is neither controlled by the IFN1 pathway nor does it regulate *MEF2A*.

Interestingly, the hyper-methylation of the CTCF I regions of CDM15/CDM13 myoblasts correlated with decreased *DMPK* antisense transcription in both CDM, suggesting that in our case, unlike the report of Nakamori et al.^27^, the transcriptional deregulation of sense/antisense transcription does not explain the more severe phenotype of CDM15.

At present, we can only speculate why CDM13 myoblasts do not activate the IFN1 response. We did not find any interspersed repeats in the CTG tract of CDM13 that could have explained the less severe phenotype. However, these myoblasts did not show signs of cellular stress during differentiation (enlarged RER and p53 upregulation) as found in CDM15. One possible explanation is that the 1800 GUG sequence in CDM13 does not form stable toxic dsRNA structures. The other possibility is that the majority of dsRNAs remain localized in the nucleus, never reaching the cytoplasm to activate the innate immune response.

In conclusion, our data show for the first time the miRNA profile of human CDM1 and control foetal myoblasts, and confirm the relevance of miRNAs in myogenesis. Our results suggest that the congenital phenotype, ascertained by triplet numbers and methylation pattern, could be more complex than expected to the extent that IFN1 activation might play a role in severe muscle pathogenesis, while yet unclarified mechanisms might lead to a less severe phenotype than predicted by current criteria (see CDM13). The results of silencing the IFN1 pathway are significant as they show rescue of aberrant myogenesis in CDM1 myoblasts; hopefully these in vitro results may lead to a better understanding of the molecular basis of this disease and identify potential targets in the search for innovative therapies.

## Methods

### Cell culture

Human myoblasts isolated from muscle biopsies obtained during autopsies of fetuses were obtained according to previously described methods^38^. Control and CDM1 myoblasts with 1800 CDM13 and 3200 CDM15 repeats, respectively, were utilized at early passages (p 4) in order to avoid senescence. Myoblasts were seeded at a density of 12,000 cells/cm^2^ in HAM’s/F10 medium supplemented with 50 µg/ml fetuin, 5 µg/ml insulin, 10 ng/ml EGF, 0.5 mg/ml BSA, and 20% FBS (Euroclone). Medium was changed every day. When cells reached about 80% confluence (40,000–50,000 cells/cm^2^), differentiation to myotubes was induced by incubating the cells into a serum-free MEM (Euroclone) supplemented with 10 µg/ml insulin and 0.5 mg/ml BSA. The number of myotubes (i.e. myoblasts with more than three nuclei) and the number of nuclei per myotube were evaluated after Giemsa staining.

### Genotyping of the CDM13 myoblasts

Genomic DNA was extracted from myoblasts using a salting-out procedure. (CTG)*n* repeat expansion sizes were determined by long-PCR (LR-PCR), followed by hybridization with a (CTG)_5_ radioactively labelled probe^39^. Primers used for LR-PCR are indicated in Supplementary Table 6. Bidirectional TP-PCR analysis was also performed to exclude the presence of interruptions at the 5′ and 3′ ends of the CTG array^5^.

### Methylation-sensitive high-resolution melting (MS-HRM)

Two hundred nanograms of DNA isolated with DNAeasy blood and tissue kit (Qiagen) were treated with EpiTech Bisulfite kit (Qiagen) according to the manufacturer's instruction. The high-resolution melting analysis was performed using the CFX96 thermal cycler (Biorad). The primers used for the HRM analysis were described in Santoro et al.^6^ with some modifications (Supplementary Table 6). The reaction mix (total volume 10 µl) contained 0.5 µM primers (Eurofin), 5 µl EpiTect HRM master mix (Qiagen), and 10 ng of treated DNA. The PCR conditions were as follows: an initial denaturation at 95 °C for 12 min, 60 cycles of 30 s denaturation at 95 °C, 30 s of annealing, and 30 s of extension at 72 °C, followed by an HRM step of 95 °C for 10 s, 50 °C for 1 min, 65 °C for 15 s, and continuous acquisition to 95 °C at one acquisition per 0.2 °C. Annealing temperatures used for UR1, UR2, UR3, DR1, and DR2 MS-HRM analyses were 63.3, 59, 63.3, 55, and 55.7 °C, respectively. For the UR3 region the HRM protocol was applied after a pre-amplification step performed with GoTaq DNA polymerase (Promega) using 20 ng bisulfite-treated DNA in 25 µl final volume with the following PCR conditions: 5 min of an initial denaturation step at 95 °C, 40 cycles of 30 s 95 °C, 30 s 58 °C, 30 s 72 °C and a final step at 72 °C for 5 min. Fully methylated and unmethylated DNA (EpiTect methylated and unmethylated human control DNA, bisulfite converted, Qiagen) was mixed to obtain the following ratios of methylation: 0%, 12.5%, 25%, 50%, 100%. In order to obtain single methylation percentage values from MS-HRM assays, rather than a range, we applied an interpolation method developed in our laboratory^40^.

### Western blot analysis

Cell lysates were prepared as previously described^4^. Samples were loaded on 10–12% polyacrylamide gels and then transferred to nitrocellulose membranes (Amersham Biosciences). The blots were blocked with 5% skimmed milk (Amersham) in TBST. Anti-ATG5 (A0856) (1:2000), anti-ATG7 (A2856) (1:1000), anti-LC3I-II (L8918) (1:1500), anti-αtubulin (T5168) (1:8000) obtained from Sigma, and anti-p53 (DAKO, M1001) (1:2000), anti-p-p70S6 kinase (p-S6k1) (Santa Cruz, sc-7984-R) (1:500) and anti-AKT (Cell Signalling, 9272) (1:1000) were used. Incubations were performed overnight at 4 °C and bands were revealed, after incubation with the recommended secondary antibody by the chemiluminescence method (Amersham) with X-OMAT film (Kodak). Densitometric analysis of developed blots was performed with the Quantity One program. Results are expressed as mean ± SD of at least three independent experiments and data analyzed by Student’s *t*-test (*P < 0.05, **P < 0.01, ***P < 0.001).

### Electron microscopy analysis

Myoblasts from each experimental group were fixed in 2.5% glutaraldehyde in 0.1 M phosphate buffer, pH 7.3, for 20 min at room temperature, washed in the same buffer, and postfixed in 1% osmium tetroxide in 0.1 M phosphate (pH 7.3) for 1 h at room temperature, and dehydrated in graduated series of ethanol. During the last dehydration step, the cells were scraped and the cell suspensions were centrifuged to obtain pellets. These pellets were then transferred into propylene oxide and embedded in PolyBed 812 (Polyscience Inc., Warrington, PA, USA) and polymerized at 60° for 72 h. Ultrathin sections (60–80 nm), obtained with a Reichert-Jung Ultracut E (Austria) equipped with a diamond knife, were collected on 200-mesh formar/carbon-coated copper grids, double stained with aqueous uranyl acetate and lead citrate, and examined with a Jeol 100 SX transmission electron microscope operating at 80 kV.

### Morphometric analysis: assessment of autophagic vacuoles

In our experimental setting, we took advantage of cellular pellets because ultrathin sections contain randomly oriented cells, which is an appropriate condition for quantitative calculation^41,42^. For ultrastructural morphometry non-serial ultrathin sections were obtained for each experimental group as described above. Ultrathin sections were examined directly at the transmission electron microscopy at 5000X magnification in order to estimate the number of autophagic vacuoles (AV) per cell. AVs were identified as double-membraned structures (compartments) containing altered cytoplasmic organelles or cytosolic material^43^. At least three grids were examined for each experimental group and 50 cells were scored for each sample. For the diameter evaluation, a minimum of 100 vacuoles were examined at each time point. The comparison among groups was performed with the multifactor analysis of variance, MANOVA. The vacuole diameter (Table 1) was determined using ImagJ. A minimum of 25 myoblasts were selected and at least 100 vacuoles were examined. The significance was determined by ANOVA.

### siRNAs transfection

CON, CDM13, and CDM15 myoblasts were transfected in six-well plate format with lipofectamine RNAiMAX (Life Technologies) using siTLR3 (TLR3 silencer selected validate siRNA, s235, Ambion), siIRF7 (IRF7 silencer selected pre-designed siRNA, s194563, Ambion), and siCT (silencer negative control, 4390843, Ambion) as control. Cells were transfected with 6 µl of lipofectamine and 25 pmol of siRNAs following the manufacturer's instruction. After 10 h (dd0) the medium was changed and cells exposed to differentiation medium for 3 days (dd3).

### Quantification of miRNAs and mRNAs (qRT-PCR)

Total RNA was extracted from 1 × 10^6^ myoblasts using the miRNeasy mini kit (Qiagen) following the manufacturer’s recommendations. One microgram of total RNA was retrotranscribed using either the miScript II RT kit (Qiagen) or the SuperScript-VILO cDNA Synthesis Kit (Invitrogen) for miRNAs or mRNAs quantification, respectively. The reverse transcription was performed following the manufacturer’s instructions. miRNAs and mRNAs were quantified with Rotor-Gene Q 2plex (Qiagen), using the miScript SYBR Green PCR Kit (Qiagen) according to the protocol indicated by the manufacturer. The relative quantification was performed using the Rotor-Gene Q Software, normalizing to the internal controls: *U6* was used for miRNAs and the geometric mean of three reference genes (*GUSB*, *TBP*, and *RSP18*)^44,45^, whose expression did not change significantly during myoblasts differentiation, was used for mRNAs. Results are expressed as mean ± SD of at least three independent experiments and data analyzed by Student’s *t*-test (*P < 0.05, **P < 0.01, ***P < 0.001). The primers used are shown in Supplementary Table 6.

The quantification of *DMPK* sense/antisense transcripts level was performed as described by Michel et al.^46^ with some modifications. Briefly, total RNAs were retrotranscribed with QuantiTect Reverse Transcription kit (Qiagen) using specific primers for sense and antisense retrotranscription. The relative levels of sense or antisense transcripts were normalized to the mean of three reference genes as explained above.

### miRNA profiling with next generation sequencing (NGS) technology (smallRNA-seq)

NGS was performed on RNA extracted from two biological replicates of CON, CDM13, and CDM15 myoblasts at day 0 and at day 3, before and after shift into differentiation medium. The cDNA libraries were constructed using TrueSeq Small RNA kit (Illumina) according to the manufacturer’s suggestions. cDNA libraries were loaded at six-plex level of multiplexing (~4 million reads per sample) into a V3 flow cell, and sequenced in single-reads mode (50 bp) on a MiSeq sequencer (Illumina).

### NGS data analysis

Pre-miRNA sequences from miRBase v.21 (http://www.mirbase.org/) were identified from raw sequences as previously described^47^ using miRExpress tool v.2.1.3^48^. The miRNomes of each sample were analyzed using Bioconductor’s package DESeq2^49^. Specifically, the original raw count data were first scaled for library size factors and regularized log transformed (rlog function) for the principal component analysis, then they were used to test for differential expression after per gene dispersion estimation. A fold change (FC) of 2 was used to select modulated miRNAs, with *P*-adjusted for multiple testing < 0.05 (Benjamini and Hochberg procedure).

### Target genes and pathways analysis

To identify genes that are targets of modulated miRNAs and to investigate altered pathways in CON, CDM13, and CDM15 myoblast cells we exploited the miRNet web-based tool (http://www.mirnet.ca)^50^. MiRNet includes a comprehensive collection of high-quality and experimentally validated miRNA-target interaction data from 11 databases. We identified targets of modulated miRNAs and performed their functional enrichment analysis for KEGG and Reactome pathways using the more suitable empirical sampling-based approach described by Bleazard et al.^51^ and implemented by miRNet. This method is more suitable for miRNAs-target genes since it takes into account the fact that each miRNA has the potential to target hundreds of different genes, while common enrichment analysis methods assume that genes are selected uniformly at random from a finite population. A *p*-value ≤ 0.005 was considered statistically significant in this case.

## Electronic supplementary material

Supplementary Data Legends

Supplementary Figures

Supplementary Tables
