# Supplementary material for: Activation of the interferon type I response rather than autophagy contributes to myogenesis inhibition in congenital DM1 myoblasts
Source: Cell Death Dis. 2018 Oct 19;9(11):1071. doi: 10.1038/s41419-018-1080-1 (PMC6195593; doi:10.1038/s41419-018-1080-1)
Supplement: Supplementary file 1 — Supplementary Data Legends [file 41419_2018_1080_MOESM1_ESM.docx]

**Supplementary Table Legends**

**Supplementary Table 1**. List of CDM15vsCON(d0)-miRNAs using |log2FC| >1, padj≤0.05 as criteria for selection. For each miRNA, base means across samples, log2 fold changes, standard errors, test statistics, p-values and adjusted p-values are reported.

**Supplementary Table 2**. List of CDM15vsCON(d3)-miRNAs using |log2FC| >1, padj≤0.05 as criteria for selection. For each miRNA, base means across samples, log2 fold changes, standard errors, test statistics, p-values and adjusted p-values are reported.

**Supplementary Table 3**. List of significantly enriched KEGG pathways (p≤0.005) among validated targets of CDM15vsCON(d0)-miRNAs.

**Supplementary Table 4**. miRNA:target interaction data of the CDM15vsCON(d0)-miRNAs and their validated targets belonging to mTOR signaling pathway. The table also reports the experimental method by which the interaction has been identified and the reference (PMID) of the paper in which it is described.

**Supplementary Table 5**. miRNA:target interaction data of the CDM15vsCON(d3)-miRNAs validated targets belonging to the “Myogenesis Pathway” (id: R-HSA-525793) in Reactome db. The table reports also the experimental method by which the interaction has been identified and the reference (PMID) of the paper in which it is described.

**Supplementary Table 6**. List of primers used in this work.
