# Supplementary material for: Activation of the interferon type I response rather than autophagy contributes to myogenesis inhibition in congenital DM1 myoblasts
Source: Cell Death Dis. 2018 Oct 19;9(11):1071. doi: 10.1038/s41419-018-1080-1 (PMC6195593; doi:10.1038/s41419-018-1080-1)
Supplement: Supplementary file 2 — Supplementary Figures [file 41419_2018_1080_MOESM2_ESM.pdf]

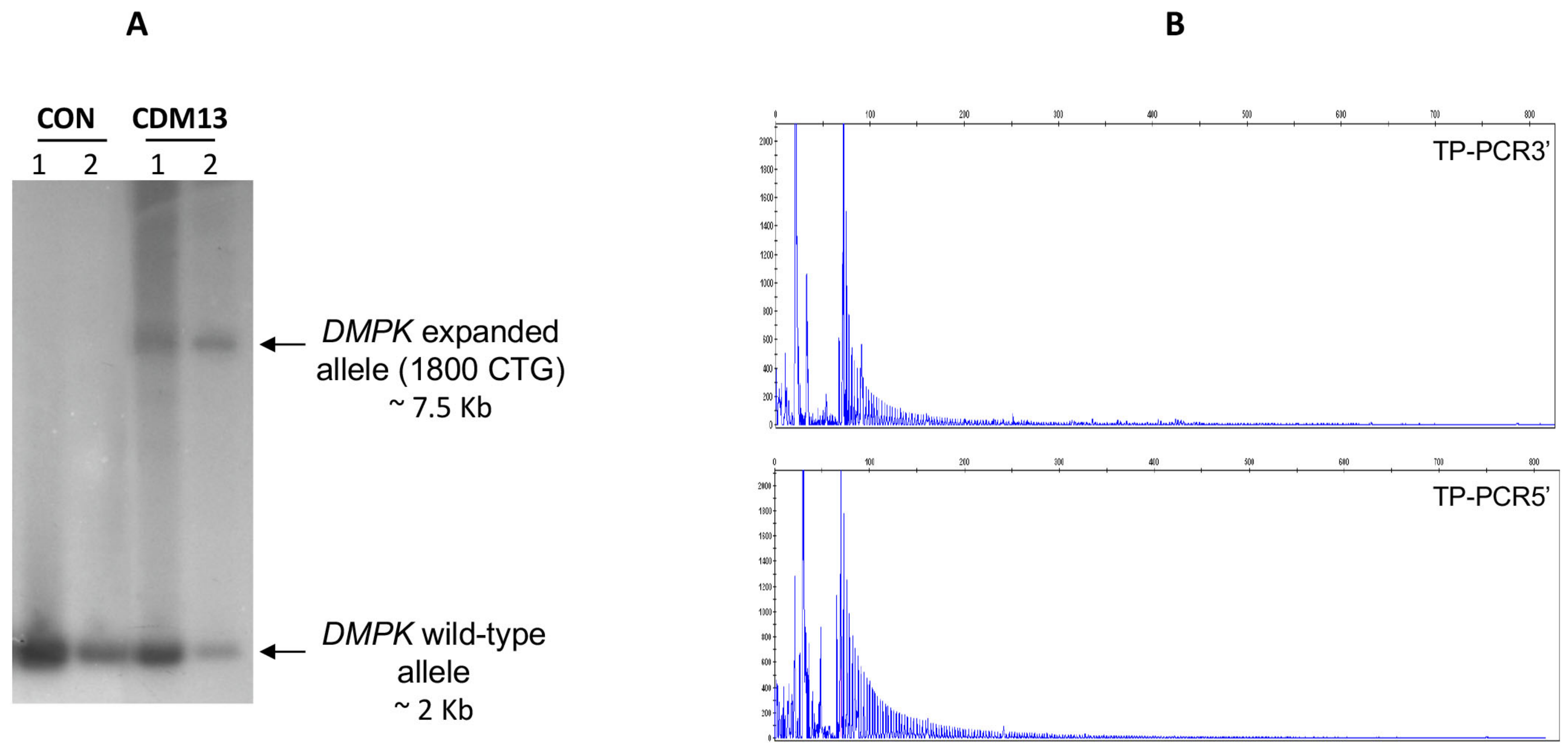

Supplementary Fig. 1. Molecular characterization of CDM13 myoblasts. **a** LR-PCR analysis of CON and CDM13 myoblasts at two different DNA concentrations (1, 2) showing the presence of a 1800 CTG expansion in CDM1 cells. **b** Triplet primed-PCR (TP-PCR) analysis of the CDM13 myoblasts at the 3' (top panel) and 5' (bottom panel) ends of the CTG array showing a regular and continuous expanded triplet peak pattern.

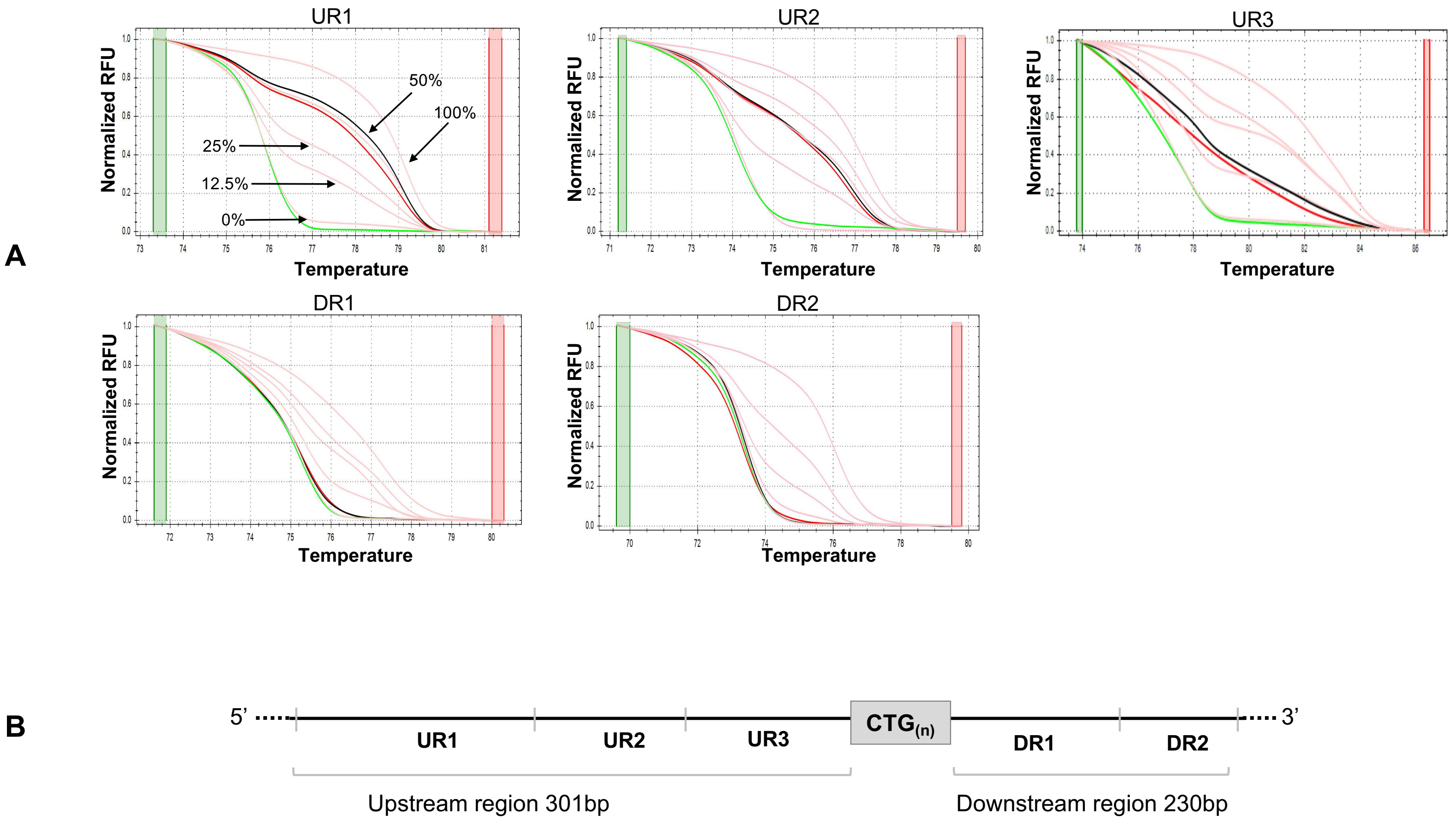

Supplementary Fig. 2. High resolution melting analysis of *DMPK* locus. **a** Melt curves profile of the region upstream (UR1-UR3, top panels) and downstream (DR1-DR2, bottom panels) the CTG repeats at the DMPK locus of CON (green), CDM13 (black), CDM15 (red) and standard template (0%, 12.5%, 25%, 50%, 100%) (pink). **b** Schematic representation of the regions analyzed at the *DMPK* locus.

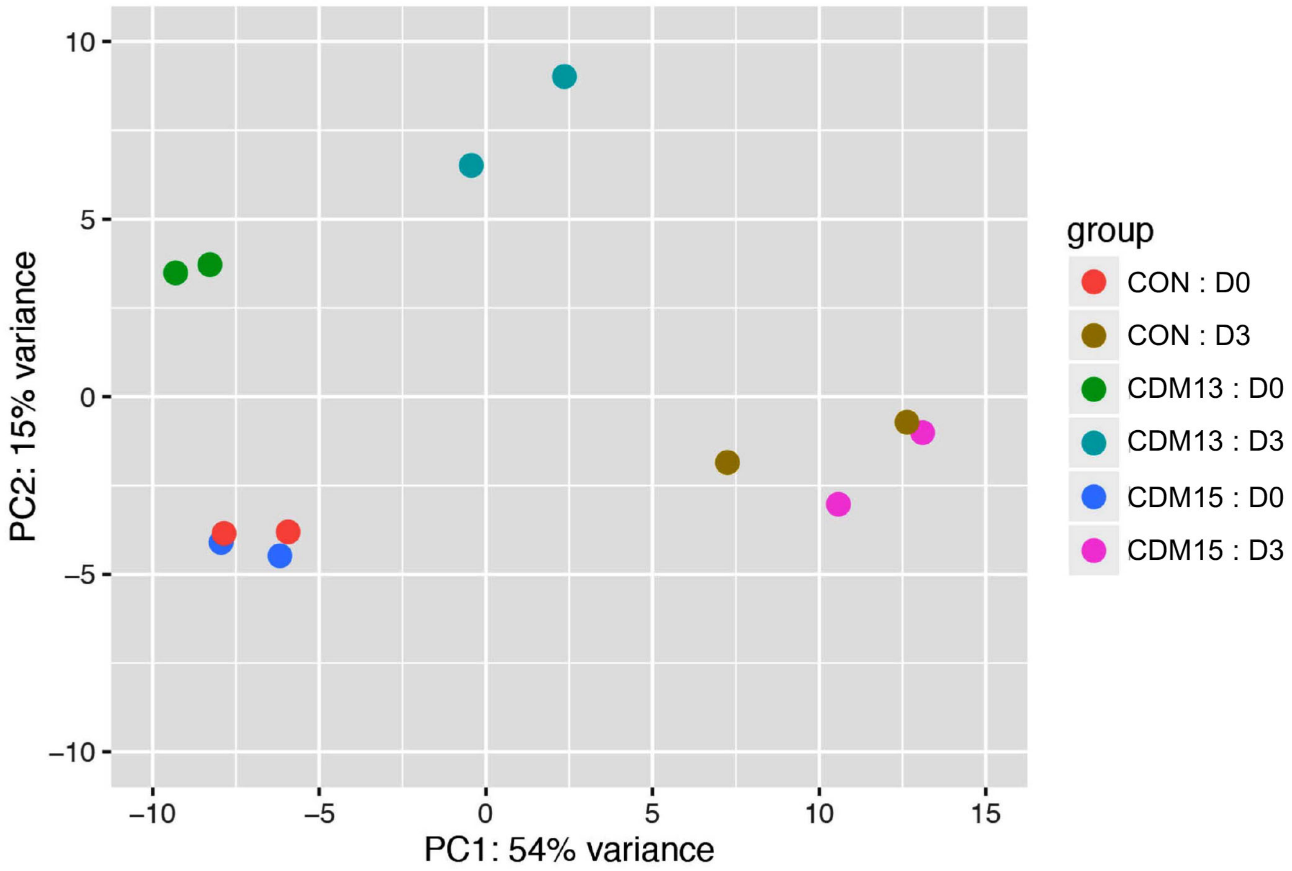

Supplementary Fig. 3. Principal component analysis (PCA) of the CON, CDM13 and CDM15 miRNA profiles. For each sample two biological replicates were performed.

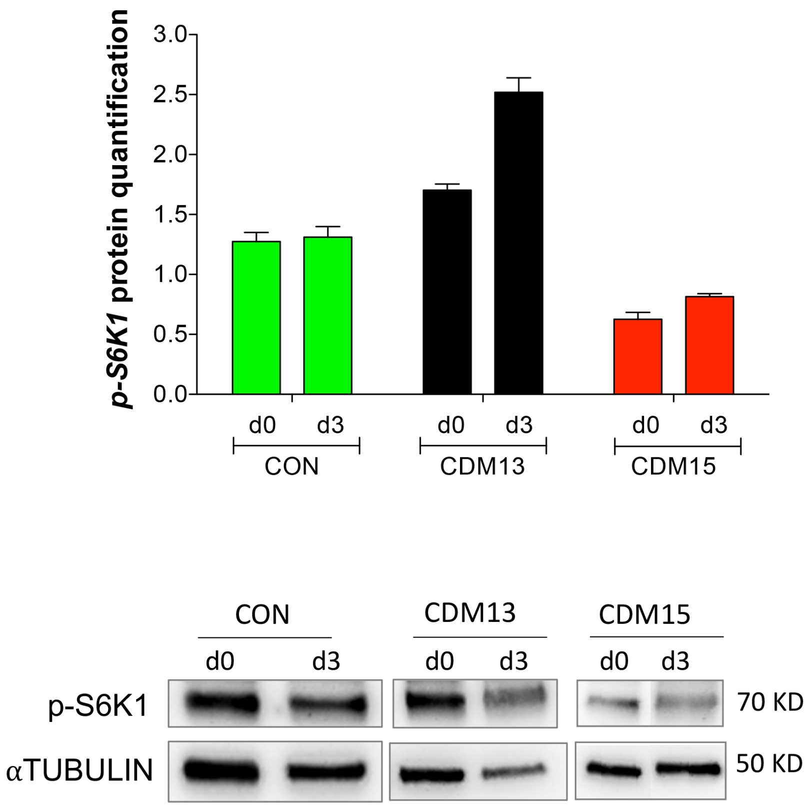

Supplementary Fig. 4. p-S6K1 protein level in CON, CDM13 and CDM15 myoblasts before (d0) and after exposure to differentiation medium for three days (d3). Data were normalized using αtubulin.

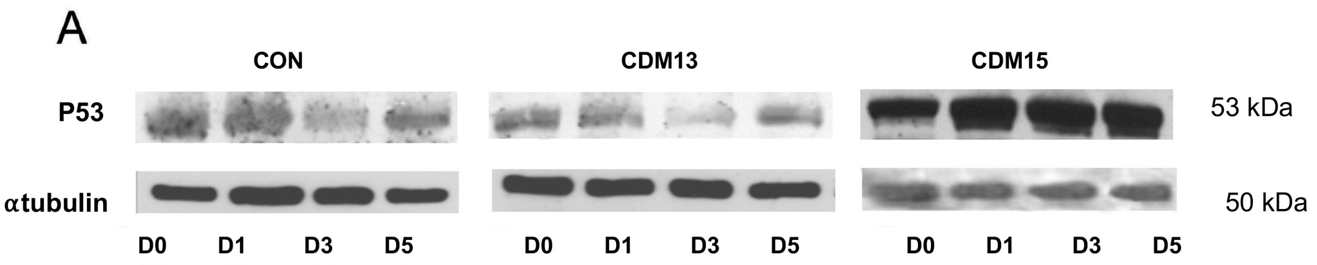

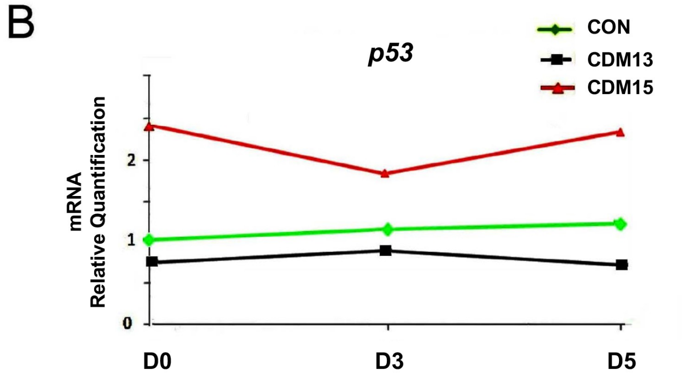

Supplementary Fig. 5. Relative p53 expression level determined by both western blot **a** and qRT-PCR **b** analysis, in CDM15, CDM13 and CON myoblasts after exposure to differentiation medium (days 0–5). The relative expression of *p53* mRNA was evaluated respect to CON at day 0. Data were normalized using the geometric mean of three reference genes (*GUSB*, *TBP* and *RPS18*).

A

	DMPK sense/antisense transcripts ratio
**CON**	0.475
**CDM13**	9.86
**CDM15**	8.74

B

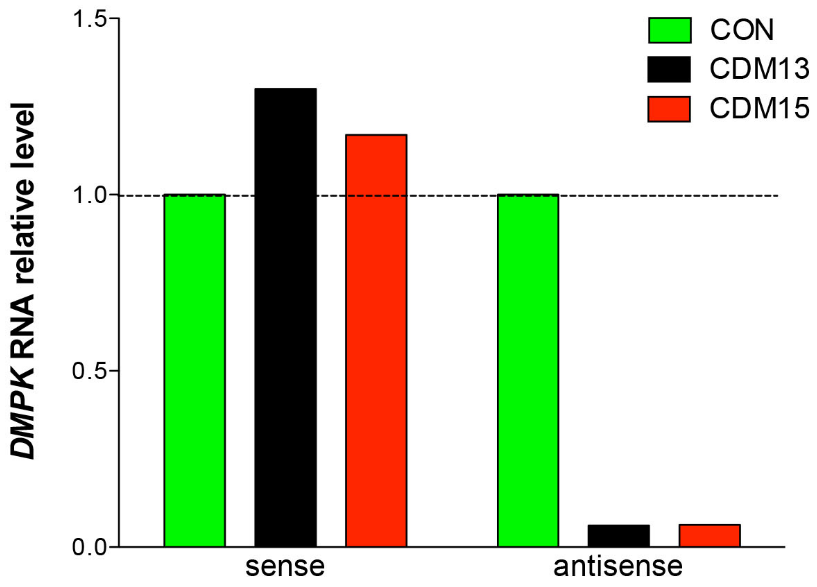

Supplementary Fig. 6. Table reporting the sense/antisense *DMPK* transcripts ratio in CDM1 and in CON myoblasts **a**. The relative quantification of sense and antisense transcripts at day 3 in CDM13 and CDM15 compared to CON is also reported **b**.

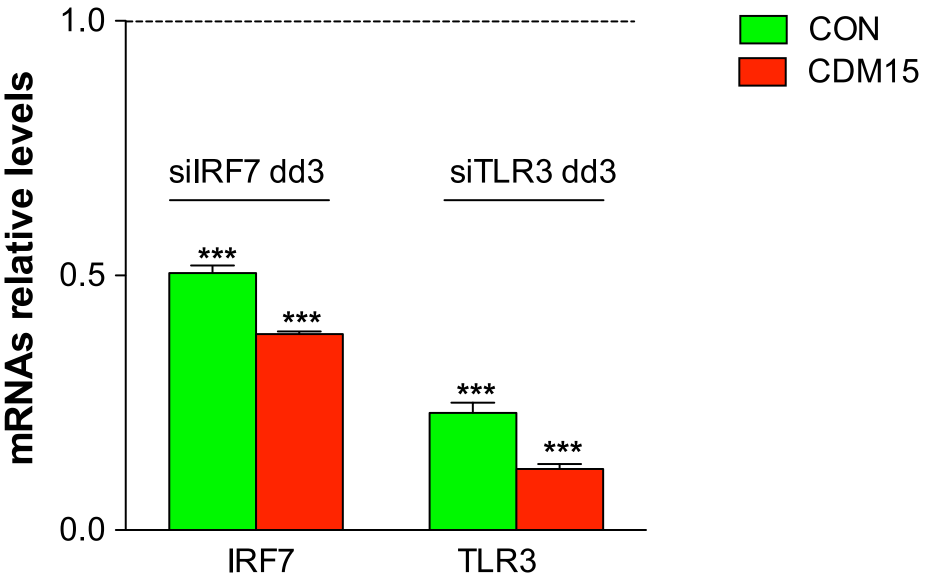

Supplementary Fig. 7. Relative IRF7 or TLR3 mRNAs level in CON and CDM15 after three days (dd3) from IRF7 (siIRF7) or TLR3 (siTLR3) inhibition versus control (siCT).
